# The Chronic Wound Microbiome: Dynamics and Treatment Response

**DOI:** 10.3390/cells15171553

**Published:** 2026-08-27

**Authors:** Ilaria Cavallo, Francesca Sivori, Massimo Francalancia, Alessandro Greco, Mauro Truglio, Fulvia Pimpinelli, Enea Gino Di Domenico

**Affiliations:** 1 UOSD Microbiology, San Gallicano Dermatological Institute, IRCCS, 00144 Rome, Italy; ilaria.cavallo@ifo.it (I.C.); massimo.francalancia@ifo.it (M.F.); mauro.truglio@ifo.it (M.T.); enea.didomenico@ifo.it (E.G.D.D.); 2 Skin Ulcer Center, Local Health Authority (ASL), 03100 Frosinone, Italy; a.greco2009@gmail.com

**Keywords:** chronic wounds, microbiome, biofilm, skin ulcers, debridement, antibiotics

## Abstract

**Highlights:**

**What are the main findings?**

- Chronic wounds harbor dynamic microbial ecosystems shaped by tissue damage, oxygen availability, host responses, biofilm organization, and antimicrobial pressure.
- Therapeutic interventions such as cleansing, debridement, topical treatments, oxygen-based approaches, and antimicrobial therapy can remodel wound microbial communities, but microbiome shifts do not automatically predict healing.

**What are the implications of the main findings?**

- Wound microbiome analysis should move beyond descriptive taxonomic profiling toward functional interpretation, integrating metagenomics with culturomics, antimicrobial susceptibility testing, biofilm assays, and clinical metadata.
- Prospective studies should determine whether microbial features predict treatment response and whether their use in clinical decisions improves outcomes beyond conventional assessment and microbiology.

**Abstract:**

Chronic ulcers require prolonged care and carry an increased risk of infection and limb loss. Sequencing studies of diabetic foot ulcers, venous leg ulcers, and pressure ulcers have revealed dynamic, patient-specific microbial communities whose composition varies with local tissue conditions and treatment exposure. This review examines changes in microbial burden and community structure during wound-bed and antimicrobial interventions and their relationships with healing. Observed shifts may contribute to therapeutic effects, reflect direct perturbation of the community, or follow tissue recovery. However, most studies are small, heterogeneous, and uncontrolled, limiting causal inference. No published randomized trial has shown that treatment selection informed by sequencing-based microbial profiling improves wound healing. A central translational challenge is to determine whether baseline microbial features reproducibly predict responses to defined interventions. Progress will therefore require prospective longitudinal studies integrating sequencing with quantitative culture, isolate phenotyping, antimicrobial susceptibility testing, biofilm assays, and standardized clinical data. Such studies should establish whether microbiome profiling adds clinically useful information to conventional wound assessment and microbiological testing.

## 1. Introduction

The study of the human microbiome has transformed our understanding of health and disease, offering critical insights into host–microbe interactions in immunity, metabolism, and tissue repair. Among the many applications of microbiome research, chronic wound care presents both a unique opportunity and a profound translational challenge. Chronic wounds, including diabetic foot ulcers (DFUs), venous leg ulcers (VLUs), and pressure ulcers (PUs), are frequently colonized by diverse and dynamic microbial communities, often organized into biofilms that contribute to inflammation, impede epithelialization, and delay healing. Numerous studies have shown that specific microbial patterns, including dominance by *Staphylococcus aureus*, *Pseudomonas aeruginosa*, or other anaerobic bacteria, correlate with poor healing trajectories and antibiotic resistance [1,2,3,4]. These microbial patterns, however, cannot be interpreted independently of the host. Glycemic control, vascular status and tissue perfusion, age, and immune competence or immunosuppression are established determinants of the wound microenvironment and healing response [5,6,7]. Sequencing and multiomic studies further indicate that glycemic control and other clinical characteristics are associated with the structure of the wound microbiome, and that infection severity is accompanied by coordinated changes in microbial function and host immune responses [1,8,9]. Systematic capture of these variables as longitudinal clinical metadata is therefore essential to distinguish host-associated variation from microbiome-specific signals and to establish the clinical relevance of microbial profiles [8,10].

Sequencing-based approaches such as 16S rRNA gene profiling and shotgun metagenomics have enabled high-resolution characterization of wound microbiota, uncovering novel associations among microbial diversity, strain-level variation, and clinical outcomes [3,11,12,13]. For instance, temporal instability in microbial communities is associated with improved healing, whereas static communities dominated by pathogenic taxa tend to persist in non-healing wounds [12]. However, these associations do not imply causality, and it remains unclear whether microbial community dynamics actively promote healing or simply reflect underlying changes in the wound environment. Moreover, functional genomic analysis has revealed extensive resistome signatures in chronic wounds, with multidrug-resistant organisms such as methicillin-resistant *Staphylococcus aureus* (MRSA) and carbapenem-resistant *Klebsiella pneumoniae* (CRKP) frequently harboring genes associated with biofilm formation, efflux pumps, and mobile genetic elements [1,14,15].

Despite these insights, the clinical utility of microbiome data in wound management remains limited. Most sequencing studies remain observational, and findings rarely guide treatment decisions or inform infection control protocols. The disconnect between microbiome science and clinical practice reflects both technical and conceptual barriers. Current workflows lack standardization in sampling, DNA extraction, and bioinformatic analysis, resulting in data that are often difficult to compare across studies or integrate into care pathways [16].

A key limitation is the inability of taxonomic profiling alone to capture microbial function, such as antibiotic susceptibility, metabolic capacity, or virulence potential, all critical parameters for clinical decision-making. Even functional predictions from metagenomic data remain inferential without experimental validation. Microbiome research has prioritized data generation over theory-building and has too often progressed independently of classical microbiology [17]. This separation has hindered the development of robust, hypothesis-driven frameworks necessary to translate microbiome knowledge into practical therapeutic applications [16,18]. The growing burden of antimicrobial resistance in chronic wound isolates has also prompted interest in alternative or adjunctive strategies, including topical essential oils and probiotic formulations, which have shown in vitro activity against key wound pathogens and may complement microbiome-informed therapeutic frameworks [19].

In contrast, culturomics, the high-throughput culture-based isolation of microbial taxa coupled with phenotypic and genomic characterization, offers a powerful, complementary approach to sequencing. Unlike NGS, culturomics enables direct functional interrogation of isolates, including antimicrobial susceptibility testing (AST), biofilm-forming ability, and metabolic profiling. It also allows differentiation between commensal and pathogenic phenotypes within a species, a distinction often obscured in taxonomic data [20]. Recent work integrating culturomics with WGS has improved the identification of resistance determinants and virulence factors, providing actionable data for clinicians [1,15,21]. However, although these integrated approaches improve biological resolution, prospective clinical studies have not yet demonstrated their incremental effect on treatment selection or healing outcomes.

Ultimately, the promise of microbiome-informed chronic wound care lies not just in identifying microbial signatures of disease but in developing functionally informed, patient-specific therapeutic strategies. This will require a fundamental shift: from passive, descriptive microbiome studies to integrated, mechanistic research frameworks that combine omics data, classical microbiology, and clinical relevance. In this review, we explore how different therapeutic strategies, wound cleansing, debridement, and antimicrobial therapies modulate the skin microbiome and influence healing trajectories. By aligning microbial dynamics with therapeutic action, we aim to clarify the potential and limitations of current approaches and advocate for a translational roadmap that redefines microbiome science in chronic wound care.

## 2. Materials and Methods

Relevant literature between January 2016 and June 2026 was identified through PubMed, Scopus, and Web of Science. The final search was conducted on 23 June 2026. Search strings combined terms related to chronic wounds, wound microbiome, diabetic foot ulcers, venous leg ulcers, pressure ulcers, biofilm, debridement, cleansing, topical treatments, antimicrobial therapy, 16S rRNA gene sequencing, fungal ITS sequencing, shotgun metagenomics, or virus-enriched metagenomics. The starting date corresponded to the earliest original chronic wound microbiome study included in the reference framework [3]. Peer-reviewed human studies assessing microbial community composition, diversity, functional potential, or temporal dynamics using 16S rRNA gene sequencing, fungal ITS sequencing, virus-enriched metagenomics, or shotgun metagenomics were eligible. Priority was given to longitudinal studies and clinically characterized cohorts reporting microbiological and wound-healing outcomes. The microbiome-focused synthesis excluded conference abstracts, studies unrelated to chronic wounds, reports lacking sufficient methodological detail, and exclusively culture-dependent investigations. Mechanistic, therapeutic, culture-based, and consensus publications were considered separately, as needed, to contextualize treatment effects and clinical implications. Given the narrative design of the review, no formal risk-of-bias assessment was undertaken. Discordant findings were retained and interpreted in light of differences in study design, wound type, sampling strategy, sequencing platform, treatment exposure, and clinical endpoint definition. The strength of the evidence was assessed qualitatively according to study design, cohort size, presence of an appropriate comparator, longitudinal follow-up, microbiological methodology, and linkage to predefined clinical outcomes. Greater interpretive weight was assigned to larger longitudinal studies with clinically characterized cohorts, whereas small uncontrolled pilot studies, cross-sectional analyses, and preclinical investigations were considered exploratory or hypothesis-generating. This evaluation was intended to contextualize the evidence rather than constitute a formal risk-of-bias assessment. Supplementary Table S1 summarizes the design characteristics, principal findings, and limitations of all primary studies.

## 3. Skin Microbiome and Clinical Relevance in Chronic Wounds

Advances in microbiome research have elucidated the spatial, temporal, and functional heterogeneity of microbial communities in chronic wounds. This heterogeneity is shaped by local environmental conditions, including tissue oxygenation, inflammation, and host immune status, which together influence the taxonomic composition, metabolic activity, and pathogenic potential of resident microorganisms [18,22]. Although the chronic wound microenvironment is shaped by multiple host and tissue factors, direct causal links between individual variables and specific microbial community configurations remain insufficiently established in sequencing-based studies and should therefore be interpreted with caution. Microorganisms are not passive contaminants but active modulators of the wound milieu. Through interactions with host immune pathways and other microbial taxa, they contribute to chronic inflammation, tissue destruction, and impaired re-epithelialization [14,23].

The healthy skin microbiome may be viewed as an adaptive functional compartment governed by host epithelial, immune, vascular, and neural signals. Within the “slave tissue” framework, commensal microbes function as an externally acquired microbial tissue whose structure and activity are constrained by human master tissues [24]. In intact skin, this control supports barrier function, colonization resistance, and immune calibration. Chronic ulcers disrupt this hierarchy. Epithelial loss, matrix degradation, impaired perfusion, altered innervation, hypoxia, and persistent inflammation weaken host regulation and create permissive niches for opportunistic dominance.

Chronic wounds, such as DFUs, VLUs, and PUs, are particularly prone to microbial perturbation, and shifts in community structure have been associated with delayed healing and increased infection risk [5,13].

Microbial colonization is ubiquitous in chronic wounds, but colonization alone does not equate to infection. A continuum from contamination to colonization and overt infection has been proposed, with progression determined by microbial load, virulence traits, and host response [5]. Colonizing organisms may exert either beneficial or detrimental effects on healing, depending on their ability to induce inflammation, evade immunity, or contribute to biofilm formation [14].

Culture-independent analyses have demonstrated that chronic wound microbial communities typically contain genera such as *Staphylococcus*, *Pseudomonas*, *Corynebacterium*, *Enterococcus*, *Streptococcus*, and *Cutibacterium* [5,13]. Compared to healthy skin, chronic wounds often exhibit reduced microbial diversity and increased dominance of pathogenic taxa, particularly *Staphylococcus aureus* and *Pseudomonas aeruginosa*. Anaerobic species are also frequently detected, particularly in DFUs, where peripheral vascular disease creates a hypoxic environment that supports obligate anaerobes such as *Finegoldia*, *Peptoniphilus*, and *Prevotella* [3,5].

In DFUs, *S. aureus* is a dominant and clinically significant species due to its adhesive properties, immune evasion mechanisms, and biofilm-forming capacity. Strains expressing high levels of staphyloxanthin, a carotenoid pigment linked to oxidative stress resistance, are enriched in non-healing wounds [1,21]. *P. aeruginosa* is also commonly isolated from DFUs, where it disrupts angiogenesis and epithelial repair through the production of cytotoxic metabolites and biofilm formation [5]. Deep tissue DFUs are often polymicrobial, with facultative and strict anaerobes contributing to recalcitrance and antibiotic tolerance [3].

VLUs, which develop in the context of venous insufficiency, generally exhibit higher microbial diversity than DFUs. Dominant taxa include *Staphylococcus*, *Streptococcus*, and anaerobes. However, bacterial richness is not consistently associated with healing, indicating that clinical outcomes are shaped by a combination of microbial, host, and environmental factors [3,13]. The presence of typical pathogens, including *P. aeruginosa*, does not uniformly predict poor healing, highlighting the need for context-specific interpretation of microbiome data [25].

PUs, often affecting immobilized or elderly individuals, share microbiological features with DFUs and VLUs. High loads of *S. aureus*, *Enterococcus faecalis*, and anaerobic bacteria are common. These wounds frequently harbor polymicrobial biofilms, which contribute to persistent inflammation, tissue necrosis, and increased susceptibility to systemic infection [5,26].

Polymicrobial interactions are central to chronic wound pathophysiology. Co-colonization by *S. aureus* and *P. aeruginosa*, for example, can result in synergistic biofilm formation, metabolic interdependence, and enhanced immune evasion [14]. Biofilms represent a key barrier to effective therapy, as bacteria embedded in the extracellular polymeric matrix exhibit reduced metabolic activity, altered gene expression, and increased tolerance to antibiotics and host immune responses [3,23]. Biofilm-associated antimicrobial tolerance results from several complementary and spatially organized mechanisms and should be distinguished from genetically inherited antimicrobial resistance [27]. The extracellular polymeric matrix can retard the penetration or sequester selected antimicrobial compounds, while steep oxygen, nutrient, and pH gradients generate physiologically heterogeneous populations containing slow-growing, metabolically inactive, or persister cells that are poorly susceptible to growth-dependent antibiotics. Biofilm growth can also induce stress response pathways, efflux systems, quorum-sensing networks, and interspecies metabolic interactions that enhance collective survival. The close proximity of microbial cells may additionally facilitate horizontal transfer of resistance determinants, whereas the matrix and biofilm architecture reduce phagocytic clearance and exposure to host immune effectors [27,28]. Consequently, conventional planktonic susceptibility testing may underestimate antimicrobial tolerance within wound-associated biofilms, which can persist after treatment and rapidly re-establish following incomplete debridement [27,29].

The spatial architecture of the wound microbiome further influences therapeutic resistance. Stratified microenvironments develop, with anaerobes localized in the hypoxic wound bed and aerobes occupying superficial layers. These gradients affect local pH, redox potential, and nutrient availability, thereby shaping microbial function and persistence [17].

Temporal dynamics are also critical. Indeed, longitudinal studies have shown that healing wounds undergo progressive microbial shifts, characterized by increased prevalence of skin commensals such as *Staphylococcus epidermidis* and *Corynebacterium* spp. and decreased abundance of anaerobic pathogens [12,30]. In contrast, non-healing wounds often exhibit temporal stability and a predominance of biofilm-forming pathogens, suggesting that microbial plasticity may indicate responsiveness to therapy [3,18].

A longitudinal 16S rRNA gene sequencing study sampled 30 clinically infected DFUs at two visits separated by 34 ± 5.8 days. Healed ulcers showed higher relative abundances of *Alcaligenes* and *Corynebacterium*, whereas worsened ulcers showed higher *Enterococcus* and *Serratia* abundance, although overall beta diversity did not differ significantly among outcome groups. PICRUSt2 analysis identified differences in predicted functional pathways, but these estimates were generated from 16S taxonomic profiles and do not directly measure microbial gene content, expression, or activity [31].

Commensal organisms such as *S. epidermidis* may exert protective effects by inhibiting pathogen colonization and stimulating host immunity [13]. *S. epidermidis* actively coordinates the skin response to injury [32]. Commensal staphylococci, including *S. epidermidis*, induced neutrophil CXCL10 signaling in skin wounds that recruited type I interferon-producing plasmacytoid dendritic cells (pDCs) and drove T cell-independent wound repair [33]. *S. epidermidis*-primed CD8^+^ T cells have a dual purpose in cutaneous immunity and wound healing [34]. In addition to regulating skin barrier function through interactions with dendritic cells, these cells maintain a poised type 2 state that can respond to injury and release type 2 cytokines to promote wound repair [32,34]. However, in chronic wounds, such beneficial communities are often diminished or displaced by more virulent taxa [5]. Notably, CXCL10-pDC and commensal-specific T-cell mechanisms have been demonstrated in murine models [33,34]; their relevance to polymicrobial human chronic ulcers remains untested.

## 4. How Do Therapeutic Interventions Influence the Composition and Dynamics of the Skin Ulcer Microbiome?

**Figure 1:**
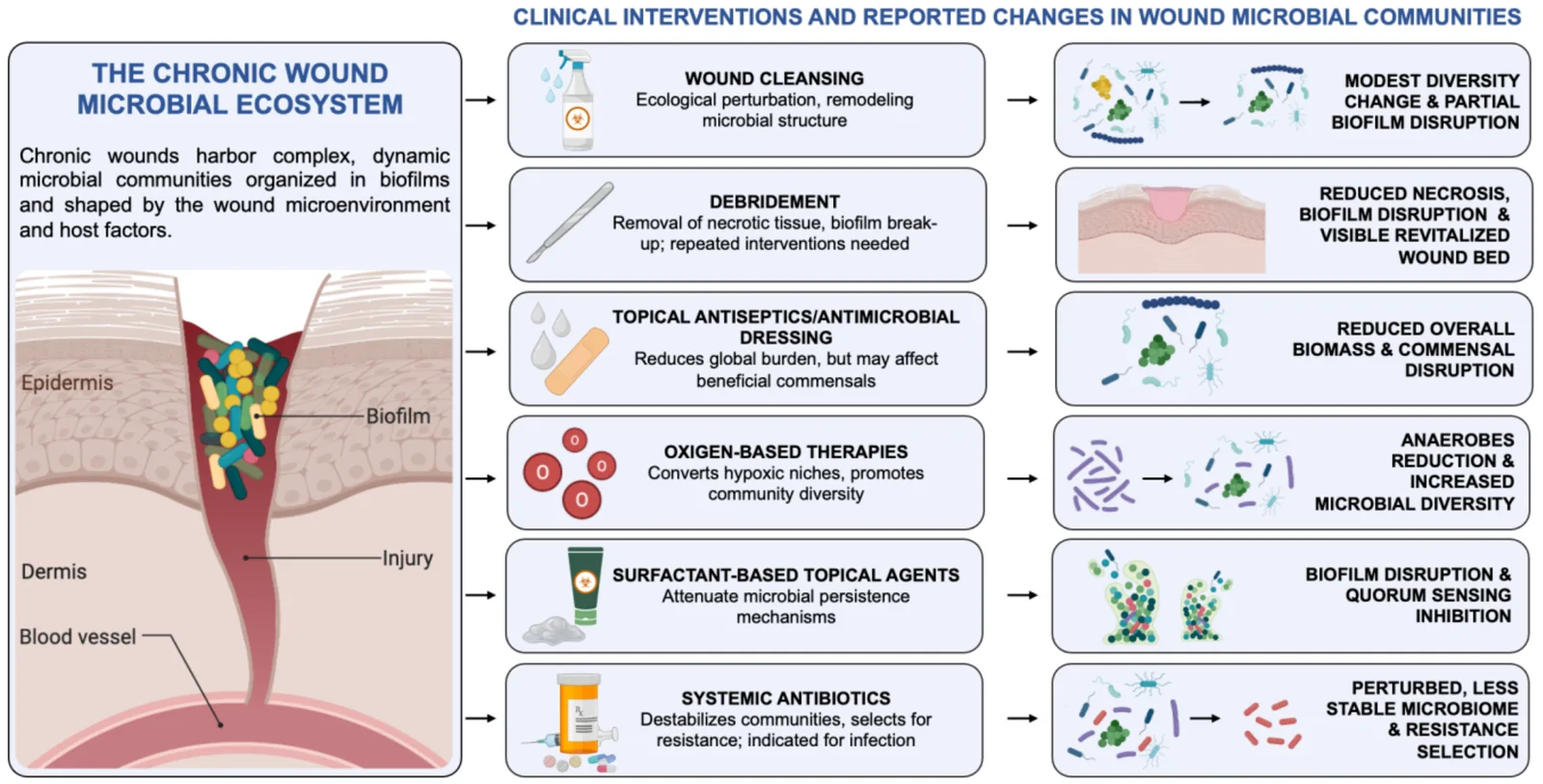
Reported and proposed effects of clinical interventions on the chronic wound microbial ecosystem. Chronic wounds may harbor polymicrobial communities, including biofilm-associated populations, shaped by tissue damage, impaired perfusion, exudate, host responses, and treatment exposure. The figure summarizes microbial changes reported or proposed in experimental, observational, and small interventional studies; these effects are context-dependent and should not be interpreted as uniformly demonstrated causal or clinical effects. Wound cleansing induces ecological perturbation and remodeling of microbial community structure, with modest changes in diversity and partial disruption of biofilms. Debridement removes necrotic tissue and mechanically disrupts biofilm reservoirs, exposing a revitalized wound bed. Topical antiseptics and antimicrobial dressings reduce overall microbial biomass and may affect commensal populations. Oxygen-based therapies modify hypoxic niches, reduce anaerobe-enriched communities, and increase microbial diversity. Surfactant-based topical agents disrupt biofilm organization and attenuate quorum-sensing-associated persistence mechanisms. Systemic antibiotics perturb community stability and select resistant populations, supporting their use in clinically infected wounds. Created with BioRender.com (https://www.biorender.com/) (accessed on 19 august 2026).

Chronic skin ulcers, including DFUs, VLUs, and PUs, represent a persistent clinical challenge, often characterized by delayed healing, recurrent infections, and high rates of antimicrobial resistance (Figure 1). A major contributor to this complexity is the multikingdom nature of the wound microbiota, encompassing diverse bacterial and fungal communities and associated viral populations that interact with the host and with one another within a dynamic, biofilm-dominated environment [13].

Evidence beyond the bacterial component of the wound microbiome remains limited but is not entirely absent. In a longitudinal study of 100 patients with diabetic foot ulcers, spanning 384 serial specimens, ITS1 sequencing detected fungal taxa in approximately 80% of ulcers, substantially exceeding the rate of culture-based detection [35]. The mycobiome was highly heterogeneous and temporally dynamic, while fungal community features were associated with antibiotic exposure, clinical complications, necrosis, and delayed healing. Selected fungal and bacterial isolates also formed mixed interkingdom biofilms, supporting the potential functional relevance of fungal–bacterial interactions [35]. More recently, metagenomic sequencing of chronic wound infections identified correlations between *Candida glabrata* and *P. aeruginosa*, and between *Malassezia restricta* and anaerobic bacteria [36]. However, these findings remain observational and require validation in etiologically homogeneous, longitudinal cohorts.

The wound virome is even less characterized and appears to be dominated by bacteriophages [37]. Virus-like particle-enriched shotgun sequencing of chronic wounds from 20 patients demonstrated greater viral diversity in wounds than in contralateral skin and identified distinct phage profiles associated with healed and non-healed wounds [37]. Complementary evidence from patients with chronic *P. aeruginosa*-infected wounds showed that the presence of filamentous Pf phages was associated with wound enlargement; mechanistic experiments suggested that Pf phages may impair keratinocyte migration through disruption of CXCL1 signaling [38]. Nevertheless, current evidence is based on few, generally small cohorts; substantial fractions of viral sequences remain unclassified; and RNA viruses and eukaryotic wound viruses are largely unexplored [37,38].

While microorganisms initially colonize all wounds, many of which originate from the host’s commensal skin flora, progression to pathogenic dominance depends on microbial virulence factors, biofilm formation, and the host’s immune status [5,39,40,41]. Historically, therapeutic interventions have centered on broad-spectrum antimicrobial strategies aimed at eradicating presumed pathogens, often with little regard for their ecological impact. However, the advent of high-resolution, culture-independent tools such as 16S rRNA sequencing and shotgun metagenomics has enabled detailed profiling of microbial communities, including strain-level identification, detection of resistance genes, and inference of functional pathways [1,30]. These approaches have highlighted that therapeutic interventions can reshape wound microbial ecosystems in a context-dependent manner, with effects influenced by microbial composition, biofilm organization, and local tissue conditions (Table 1). Rather than uniformly eradicating microorganisms, emerging strategies aim to selectively modify microbial communities while preserving functions that may support tissue recovery. This ecological perspective underscores the need to integrate microbiological characterization with clinical parameters to guide more targeted wound management strategies [5,18]. Thus, therapeutic modulation of the wound microbiome should be interpreted as an ecological intervention rather than as a simple antimicrobial event. Clinically effective treatment may require disrupting stable, non-healing microbial states, reducing protected anaerobic and biofilm-associated niches, and promoting dynamic community trajectories compatible with tissue repair.

### 4.1. Wound Cleansing

In a pre/post analysis of 11 patients with chronic VLUs, wound cleansing with hypochlorous acid- or a chlorhexidine-based soap solution was assessed for its impact on microbiota composition [42]. High-throughput 16S rRNA sequencing revealed polymicrobial communities before cleansing, often dominated by *Proteus*, *Pseudomonas*, *Morganella*, or *Providencia*. Although species richness and evenness did not change significantly at the cohort level, cleansing consistently reduced the dominant genus’s relative abundance in each wound, resulting in measurable shifts in community composition. These effects were highly patient-specific, reflecting the substantial interpersonal variability of VLU microbiomes. Culture-based data supported a reduction in viable bacterial load following cleansing [42]. A recent work characterized the resistance traits of microorganisms isolated from CPU and evaluated their susceptibility to antimicrobial cleansing agents. In this in vitro study, a panel of 20 isolates, including CRKP, multidrug-resistant *Acinetobacter baumannii* (MDRAB), MRSA, and *Candida albicans*, was analyzed for antimicrobial resistance genes (ARGs), disinfectant resistance genes (DRGs), and biofilm-forming capacity [43]. Whole-genome sequencing revealed a high burden of ARGs in CRKP and MDRAB, as well as extensive biofilm-forming potential across all isolates. Sodium hypochlorite (NaOCl) exhibited potent antimicrobial activity against both planktonic and biofilm-associated cells at significantly lower concentrations than those typically used in commercial preparations [43].

Overall, the findings suggest that wound cleansing does not simply reduce diversity but rather remodels the microbial structure, potentially destabilizing dominant taxa and disrupting biofilm communities. Such controlled perturbation of the wound microbiome may contribute to improved healing outcomes, consistent with broader evidence that dynamic microbial communities are less conducive to chronicity [14]. However, the 11-patient analysis supports an immediate, patient-specific perturbation after cleansing, but it had no untreated comparator and did not assess whether the changes persisted or improved healing [42]. The pressure-ulcer study evaluated only 20 cultured isolates in vitro [43]. Neither dataset establishes that cleansing-induced community change mediates a clinical benefit; controlled longitudinal studies linking viable microbial burden, community dynamics, and prespecified healing outcomes are required.

### 4.2. Topical Treatments

Direct human evidence that topical interventions remodel the wound microbiome and improve healing remains sparse. Silver-, iodine-, and oxygen-based treatments have antimicrobial or antibiofilm activity, but broad-spectrum activity may also affect commensal organisms [44,45]. A recent pilot study on topical oxygen therapy (TOT) in chronic DFUs showed that continuous oxygen delivery can shift the wound microbiota from anaerobe-dominated communities toward a more diverse flora enriched with aerobes and facultative anaerobes [46]. This microbial transition was observed in five out of six ulcers that healed over 8 weeks, suggesting that oxygenation may promote a microbiome profile more conducive to healing by disrupting chronic biofilm-associated dysbiosis. In contrast, the single non-healing ulcer maintained a stable, anaerobe-rich microbiome throughout the study, despite standard care and TOT. However, the study’s conclusions are limited by its small sample size (*n* = 6), which precluded robust statistical analysis and the identification of keystone microbial taxa associated with healing [46].

Surfactant-based gels applied to DFUs reduced total microbial burden in 7 out of 10 patients, but community changes were heterogeneous; *Corynebacterium*, *Streptococcus*, and *Staphylococcus* spp., persisted or increased in some wounds [47]. These changes were individualized and did not always correlate with overall bacterial load. Without a concurrent control arm and with only 10 participants, these results support biological activity but not clinical efficacy or a durable shift toward a healing-associated microbiome.

Additional evidence indicates that topical antimicrobials, such as enzymatic debriders and antimicrobial gels, not only reduce biofilm biomass but also suppress microbial gene expression associated with quorum sensing, antimicrobial resistance, and biofilm formation, thereby modifying the functional ecology of the wound microbiota [48].

Silver-based dressings effectively reduce microbial burden and bacterial biomass, but prolonged use may have off-target effects, including delayed keratinocyte migration and impaired re-epithelialization [44,49,50,51]. Furthermore, evidence on their capacity to modulate the composition and dynamics of wound-associated microbial communities remains scarce. Iodine-based treatments exhibit strong antimicrobial effects, though their impact on microbial diversity and host cells remains under investigation [52]. Topical probiotics have emerged as a promising adjunctive therapy to restore microbial balance and promote wound healing. The topical application of *Lactobacillus*-containing formulations has been shown to reduce pathogenic colonization, enhance epithelial barrier integrity, and modulate local immune responses, contributing to accelerated wound closure and reduced inflammation [53]. Evidence that topical agents induce durable and clinically beneficial restructuring of the wound microbiome remains limited. Small cohorts, short follow-up periods, and the general absence of longitudinal sequencing currently preclude drawing conclusions about their long-term ecological effects.

### 4.3. Debridement

Debridement is a cornerstone of chronic wound management. It is essential for removing necrotic tissue, slough, and senescent cells, as well as mechanically disrupting bacterial biofilms. This process not only reduces microbial burden but also creates a metabolically active wound bed that supports granulation, angiogenesis, and re-epithelialization [54,55]. In addition to these physiological benefits, debridement exerts a selective pressure on the wound microbiome. Longitudinal metagenomic studies in DFUs have demonstrated that serial sharp debridement is associated with a progressive decline in anaerobic taxa such as *Anaerococcus*, *Finegoldia*, and *Porphyromonas*, as well as decreased species richness and diversity in healing wounds. Conversely, wounds that fail to heal retain higher levels of anaerobic and proteolytic organisms and display limited microbial shifts over time, suggesting ecological resistance to debridement [1]. However, the impact of debridement on microbial community composition is not immediate. Cross-sectional studies have shown that a single sharp debridement produces only modest changes in the taxonomic structure of the wound microbiota, with pre- and post-debridement profiles remaining largely similar [2]. Importantly, wounds with baseline dominance by facultative anaerobes such as *Enterobacter* have been associated with poor healing trajectories [2]. These observations suggest that multiple repeated interventions may be required to shift the microbial ecology toward healing. Moreover, debridement alone is insufficient to eliminate biofilm communities, which can rapidly reform within 24 h, often with enhanced antimicrobial tolerance mediated by extracellular polymeric substances, metabolic dormancy, and expression of resistance genes [39,40]. In a longitudinal DFU cohort, patients received standardized care including sharp debridement, offloading, and regular follow-up. Within this controlled therapeutic setting, microbial communities displayed non-random transitions between community types, and the frequency of these transitions was associated with healing. This supports a model in which repeated wound care can destabilize persistent microbial configurations and create conditions permissive for tissue repair [12]. At the same time, early microbiome changes after standard care may be limited. A previous study examined 10 patients with vascularized neuropathic DFUs who were treated with initial sharp debridement and offloading, sampling the wound bed, wound edge, and adjacent skin at baseline and after one week. Wound beds harbored more distinct bacterial phylotypes than wound edges or adjacent skin, indicating marked spatial compartmentalization within DFUs. However, one week of standard care did not significantly change microbiome diversity or global taxonomic composition. These data suggest that a single or short-course intervention may be insufficient to remodel established wound communities, particularly when anaerobic or biofilm-associated taxa occupy protected niches within the wound bed [56]. Indeed, the same study also identified baseline microbial features associated with subsequent healing. Increased abundance of Gram-positive anaerobic cocci, particularly *Peptoniphilus*, was associated with impaired healing, whereas taxa such as *Corynebacterium* and *Arthrobacter* were more associated with wounds progressing toward closure [56]. These findings nominate baseline community features as candidate prognostic markers but do not establish prediction of debridement response or support taxon-directed treatment. Taken together, these studies indicate that debridement should be interpreted as an ecological intervention whose success depends on repeatedly disrupting persistent microbial structures and removing necrotic and hypoxic niches, creating conditions that support tissue repair. Adjunctive strategies, such as antimicrobial dressings, topical antiseptics, or enzymatic agents targeting biofilm architecture, are essential to prevent recolonization. Shifting the wound microbiome away from anaerobic dominance and persistent biofilms, while preserving beneficial skin commensals, may ultimately improve healing outcomes and reduce chronicity.

### 4.4. Antimicrobial Therapies

Chronic wounds pose a major clinical challenge, largely due to their persistent polymicrobial colonization, high prevalence of antimicrobial resistance, and frequent treatment failures. Insights from metagenomic analyses underscore the need for antimicrobial therapies that not only eliminate classical pathogens but also modulate complex microbial communities, overcome resistance mechanisms, and account for the dynamic ecological interactions within the wound microbiome. In this framework, antimicrobial efficacy should be interpreted not only as pathogen suppression but also as the capacity to reshape adverse microbial configurations, reduce biofilm-associated tolerance, and support wound trajectories compatible with tissue repair.

Historically, antimicrobial approaches in chronic wound care relied heavily on culture-based diagnostics to identify pathogens and guide systemic or topical antibiotic use [57]. However, culture-dependent methods systematically underestimate microbial diversity and fail to detect fastidious, anaerobic, or biofilm-associated organisms, which play a central role in delayed healing [13]. Molecular analysis of infected DFUs has confirmed that sequencing can reveal broader polymicrobial structures than conventional culture, including anaerobic and low-abundance taxa that may contribute to persistence through cooperative interactions within the wound niche [58,59]. This limitation has contributed to the widespread empirical use of antibiotics, often in the absence of clinical infection, thereby promoting antibiotic resistance and disrupting the beneficial components of the skin microbiota [12,13].

One of the most critical insights into wound microbiology is the recognition that microbial community dynamics, rather than the presence of a single pathogen, may predict healing outcomes. A longitudinal study of DFUs demonstrated that stable microbial communities were associated with poor wound healing, whereas microbiome instability and compositional shifts were associated with improved wound resolution [12]. Consistent with this concept, longitudinal profiling of diabetic skin and foot ulcers showed marked inter-patient variability and recurrent temporal shifts in dominant taxa, indicating that DFUs do not converge toward a single universal microbiome state [51]. This dynamic perspective challenges traditional paradigms and suggests that effective antimicrobial therapy must be tailored not only to eliminate pathogens but also to perturb or remodel microbial consortia in a beneficial direction. Systemic antibiotic exposure destabilized wound microbial communities, as reflected by increased inter-visit weighted UniFrac distances. Antibiotics administered specifically for the study ulcer produced greater community disruption than antibiotics prescribed for other indications, and the combined presence of antibiotic exposure and ulcer complications had an additive disruptive effect on community structure [12]. However, this perturbation did not simply correspond to a predictable increase in diversity or a consistent depletion of specific pathogenic taxa [12]. These findings are clinically important because they suggest that antibiotic-induced microbiome destabilization is not necessarily equivalent to therapeutic restoration. In non-infected DFUs, antibiotic therapy has not been shown to improve healing, and indiscriminate exposure may select resistant organisms while disrupting potentially beneficial microbial networks [60]. Thus, systemic antibiotics should be conceptualized as targeted tools for clinically infected wounds rather than general microbiome-modulating agents for chronic ulcers.

This interpretation is consistent with the observation that conventional culture often underestimates microbial diversity, particularly of anaerobes and fastidious organisms, and that antibiotic prescribing based solely on culture or on nonspecific signs of colonization may be poorly aligned with the true wound ecosystem [61]. Thus, the clinically relevant wound bioburden should be understood across three dimensions: microbial load, microbial diversity, and the presence of pathogenic organisms, as well as the need for longitudinal sampling and integration of clinical metadata to distinguish benign colonization from problematic microbial configurations.

Future studies should determine whether microbiome profiling can add useful information to clinical assessment, culture, and phenotypic susceptibility testing when selecting antimicrobial therapy. This is especially relevant in wounds dominated by anaerobes, *S. aureus*, *P. aeruginosa*, or polymicrobial biofilms, where conventional susceptibility testing may underestimate the resilience of sessile communities and fail to predict clinical response.

Antibiotic resistance is a pressing concern in chronic wound management. A metagenomic study of DFU tissues revealed a resistome comprising 229 antibiotic resistance gene (ARG) subtypes, including core genes associated with multidrug resistance, tetracyclines, beta-lactams, and macrolide–lincosamide–streptogramin classes [15]. Mobile genetic elements such as plasmids and transposons, frequently identified alongside ARGs, indicate the potential for horizontal gene transfer within the wound microbiome, thereby exacerbating resistance development. Moreover, systemic antibiotic therapy has been shown to destabilize microbial communities without necessarily reducing pathogenic abundance or improving diversity [1,12], further underscoring the need for more targeted, microbiome-conscious strategies.

Biofilm formation represents a major obstacle to antimicrobial efficacy in chronic wounds. Biofilms confer antibiotic tolerance through multiple mechanisms, including reduced antimicrobial penetration through the extracellular polymeric matrix, metabolic heterogeneity with the emergence of slow-growing or dormant persister cells, biofilm-specific changes in gene expression, and cooperative stress responses within polymicrobial communities [1,21]. Together, these mechanisms reduce antimicrobial efficacy without necessarily reflecting genetically encoded resistance. In DFUs, *S. aureus* and *P. aeruginosa* are commonly detected as dominant biofilm-formers, often coexisting in structured communities that enhance survival and immune evasion. Specifically, wounds containing biofilm-associated *S. aureus* exhibited increased abundance of genes associated with antibiotic resistance and persistence [1,62]. This finding suggests that standard susceptibility testing may underestimate the resilience of biofilm-embedded bacteria, leading to inappropriate therapeutic choices. Crucially, conventional susceptibility assays and taxonomic metagenomic profiling alone do not capture these tolerance mechanisms, highlighting the need to integrate biofilm phenotyping and functional culturomics into the interpretation of resistance signatures identified in chronic wound sequencing data.

A promising frontier is microbiome-based therapies. Commensal microbes may exert protective effects by competitively excluding pathogens, producing antimicrobial peptides, or modulating local immune responses [23,63]. Restoration of microbial diversity through probiotic or prebiotic approaches is conceptually appealing but remains underexplored in the context of chronic wounds [39]. In the future, targeted application of beneficial skin commensals, engineered or naturally occurring, may help reestablish microbial balance and promote healing.

Another avenue involves employing microbial signatures for diagnostic and therapeutic guidance. For example, certain microbial taxa or ARG profiles may predict nonhealing trajectories or identify candidates for specific interventions. Previous work showed that ARG abundance was closely correlated with both microbial community structure and the presence of mobile genetic elements [15]. This suggests that resistome profiling, integrated with metagenomic sequencing, could inform personalized antimicrobial therapy, especially in high-risk or treatment-refractory wounds.

Despite these advances, significant challenges remain. Translating microbiome research into clinical practice requires standardization of sampling methods, consensus on relevant endpoints, and integration with existing wound care protocols. Additional barriers include heterogeneity in sequencing depth, bioinformatic pipelines, reference databases, tissue versus swab sampling, and the limited capacity of bulk sequencing to resolve spatially organized biofilms. Moreover, the ethical implications of manipulating microbial communities, particularly in immunocompromised patients, require careful consideration. Nonetheless, the paradigm is shifting from indiscriminate antimicrobial use toward precision microbiome-modulating strategies.

In conclusion, antimicrobial therapy in chronic wound care must evolve to address the complexities of the wound microbiome. Traditional antibiotic regimens, while necessary in cases of overt infection, often fall short of addressing the polymicrobial, biofilm-rich nature of chronic wounds and may inadvertently worsen outcomes by selecting for resistance and disrupting the microbiome. Culture-independent profiling, combined with biofilm-targeted and microbiome-sparing interventions, holds promise for transforming wound care. As our understanding deepens, therapeutic strategies must be reimagined to not only suppress pathogens but also to restore microbial balance and support the host’s healing response.

**Table 1:** Therapeutic modulation of the chronic wound microbiome: mechanisms, microbial effects, and translational implications.

Therapeutic Strategy	Main Microbial/ Ecological Effect	Functional or Clinical Interpretation	Key Limitations/Open Questions	References
**Wound cleansing**	Reduces viable microbial burden and can decrease the relative abundance of dominant taxa without necessarily altering global richness or evenness.	Cleansing should be interpreted as a controlled ecological perturbation that destabilizes dominant microbial populations and may reduce planktonic and biofilm-associated biomass.	Effects are highly patient-specific; optimal cleansing agents and timing remain insufficiently standardized.	[14,42]
**Topical** **antiseptics and antimicrobial dressings**	Reduce microbial burden and biofilm biomass; may exert selective pressure on wound communities and alter the abundance of dominant bacterial groups.	Useful for reducing pathological bioburden and local microbial virulence, particularly when integrated into structured wound hygiene protocols.	Broad-spectrum activity may affect beneficial commensals and host cells; long-term effects on microbial ecology, epithelial repair, and recurrence remain poorly characterized.	[44,45,64,65]
**Debridement**	Removes necrotic tissue, slough, senescent cells, and part of the microbial biofilm; repeated debridement may reduce anaerobic and proteolytic taxa.	Acts as both a mechanical and ecological intervention, creating a more permissive wound bed for granulation, angiogenesis, epithelialization, and microbial succession toward healing states.	A single debridement may produce limited short-term changes in community composition; biofilms can rapidly re-establish; efficacy depends on repetition and combination with adjunctive antimicrobial or antibiofilm strategies.	[1,2,12,54,55,56]
**Systemic** **antibiotics**	Can destabilize microbial communities and alter inter-visit community distances; may reduce target pathogens but can also select resistant populations and disrupt host-compatible microbial networks.	Should be reserved for clinically infected wounds and interpreted as targeted antimicrobial therapy rather than general microbiome modulation.	Antibiotic-induced microbial destabilization does not necessarily translate into tissue repair; standard susceptibility testing may underestimate tolerance in biofilm-associated communities.	[1,12,13,15]
**Surfactant-based topical agents**	Reduce total microbial burden and can modify community structure; may suppress microbial pathways related to quorum sensing, antimicrobial resistance, and biofilm formation.	These agents may shift treatment from simple killing toward functional attenuation of microbial persistence mechanisms.	Evidence remains limited by small cohorts, proof-of-concept designs, and incomplete longitudinal microbiome assessment.	[48,58]
**Oxygen-based therapies**	Increase local oxygen availability and may shift anaerobe-dominated communities toward more diverse communities enriched in aerobic and facultative anaerobic taxa.	Oxygenation may counteract hypoxic niches that support anaerobic persistence and mature polymicrobial biofilms.	Current evidence is based on small studies; it remains unclear which microbial changes are causal drivers of healing rather than markers of improved tissue oxygenation.	[5,13,46]

## 5. From Microbial Dynamics to Therapeutic Action

Microbial data may help characterize treatment response, but evidence that they can select an effective intervention remains insufficient. Wound communities are shaped by tissue oxygenation, necrosis, exudate, immune pressure, antimicrobial exposure, and repeated wound-bed care. Therapies such as cleansing, debridement, topical antiseptics, systemic antibiotics, and advanced dressings should therefore be interpreted as ecological perturbations. These interventions can either disrupt persistent microbial states or impose selective pressures favoring tolerant, resistant, or biofilm-associated populations. This distinction is clinically important. Longitudinal studies indicate that healing wounds often undergo microbial reconfiguration, whereas non-healing wounds frequently retain temporally stable communities dominated by pathogens or anaerobic taxa [1,18]. However, microbiome shifts are not intrinsically beneficial. Antibiotic-induced destabilization, reduced diversity, or depletion of dominant taxa does not necessarily translate into tissue repair, particularly when microbial changes are uncoupled from wound-bed viability, perfusion, immune resolution, and epithelial regeneration [13,56]. Microbiome measurements should therefore be interpreted primarily as readouts of treatment-associated change; their value as response biomarkers and predictors of treatment response requires prospective validation.

A major obstacle to this translational transition is the limited capacity to identify clinically relevant biofilm and anaerobic microbial activity in routine practice. Although biofilm is widely recognized as a central driver of chronic wound persistence, no validated point-of-care diagnostic test currently allows reliable detection, quantification, or longitudinal monitoring of biofilm burden in vivo [58,66]. Clinical assessment therefore relies on indirect indicators such as excessive exudate, recurrent slough formation, delayed granulation, and failure of standard antimicrobial strategies. Similarly, culture-independent sequencing approaches consistently detect anaerobic microorganisms, particularly in diabetic foot ulcers and hypoxic wound environments, yet their precise functional contribution to impaired healing remains incompletely understood [3,5,13]. Their presence may reflect active pathogenic involvement, adaptation to hypoxic niches, or the structural organization of mature polymicrobial biofilms, rather than direct causality. Resolving these uncertainties will require prospective studies integrating sequencing with quantitative culture, isolate phenotyping, antimicrobial susceptibility testing, biofilm assays, spatial microbiology, and standardized clinical data.

These limitations support the emergence of wound hygiene as a practical framework for structured wound-bed management. Rather than focusing exclusively on pathogen eradication, wound hygiene conceptualizes cleansing, debridement, wound-edge management, and the appropriate use of topical antimicrobials as repeated, structured ecological interventions aimed at preventing biofilm re-establishment and maintaining a wound environment conducive to tissue repair [64]. Within this framework, the goal is not sterilization of the wound, an outcome that is neither achievable nor biologically desirable, but rather the control of microbial virulence, biofilm resilience, and pathological bioburden while limiting unnecessary antimicrobial exposure. This approach aligns with longitudinal observations linking healing to dynamic microbial restructuring rather than complete microbial eradication [1,12]. In this perspective, therapeutic success should therefore be defined by effective control of microbial burden and activity, rather than by the elimination of specific taxa.

Consequently, integrating microbial profiles with clinical data and treatment history may improve biological stratification, but this approach has not been prospectively validated. A central translational challenge is to determine whether baseline microbial features reproducibly predict responses to defined interventions. Most available studies are small, heterogeneous, and uncontrolled. No published randomized trial has therefore shown that treatment selection informed by sequencing-based microbial profiling improves wound healing. Prospective longitudinal studies should first establish the reproducibility and incremental value of candidate profiles. Randomized strategy trials should then determine whether incorporating validated profiles adds clinically useful information to conventional wound assessment and microbiological testing.

## 6. Conclusions

From a microbiological perspective, microbiome studies have substantially advanced understanding of chronic wounds by showing that they comprise dynamic, heterogeneous microbial ecosystems rather than static assemblages of individual pathogens. Sequencing-based analyses have revealed previously underappreciated anaerobic and low-abundance taxa, strain-level and functional variation, resistome potential, and patient-specific community changes associated with healing, infection, and therapeutic intervention. These findings have provided a richer biological framework for interpreting wound chronicity and may support the identification of candidate microbial signatures for future diagnostic development and therapeutic stratification. Integrating these community-level observations with culture, isolate-level phenotyping, and established principles of microbial physiology and ecology should further strengthen their functional and mechanistic interpretation. Nevertheless, the available human evidence remains quantitatively uneven and predominantly observational, limiting the ability to distinguish microbial changes that influence wound trajectories from those that reflect alterations in tissue state, host physiology, or treatment exposure. No randomized trial has yet shown that assigning therapy based on a microbiome profile improves healing; microbiome profiles therefore cannot yet serve as standalone clinical decision tools. Clinical translation will require standardized longitudinal sampling, validated measures of viable microbial burden and biofilm activity, integration with host and wound characteristics, and prospective controlled trials demonstrating that microbiome-informed management changes therapeutic decisions and improves prespecified clinical outcomes (Figure 1). Until such evidence is available, molecular microbial profiling should complement clinical assessment of infection, perfusion, tissue viability, and treatment response, together with culture-based microbial identification and phenotypic antimicrobial susceptibility testing.

## Data Availability

No new data were created or analyzed in this study. Data sharing is not applicable to this article.

## Supplementary Materials

The following supporting information can be downloaded at: https://www.mdpi.com/article/10.3390/cells15171553/s1.
