# Supplementary material for: The Chronic Wound Microbiome: Dynamics and Treatment Response
Source: Cells. 2026 Aug 27;15(17):1553. doi: 10.3390/cells15171553 (PMC13565319; doi:10.3390/cells15171553)
Supplement: Supplementary file 1 [file cells-15-01553-s001.zip › cells-4429269-supplementary.pdf]

	Study design / evidence domain	Wound type or experimental model	Sample size / biological material	Sampling and microbiological / analytical methods	Key findings relevant to this review
**[1] Kalan et al. (2019)**	Prospective longitudinal human cohort with embedded culture-based phenotyping, isolate genomics, and diabetic-mouse wound experiments; follow-up to 26 weeks.	Neuropathic diabetic foot ulcers (DFUs); db/db mouse excisional wounds for selected isolates.	100 participants; 384 serial wound specimens. Shotgun-metagenomic subset: 195 specimens from 46 participants. Additional isolate-level and small murine experiments.	Serial wound sampling during standardized care; 16S rRNA gene profiling, shotgun metagenomics, bacterial culture, isolate whole-genome sequencing and phenotyping, and murine wound infection models.	Species- and strain-level variation was associated with healing and complications. S. aureus strains produced different wound phenotypes; anaerobe depletion after debridement was most evident in favorable outcomes, whereas resistance genes were widespread.
**[2] Verbanic et al. (2020)**	Single-center observational pre/post study of one sharp-debridement episode, with 6-month ascertainment of healing.	Heterogeneous chronic wounds in outpatients; paired healthy skin sampled as a within-patient comparator.	20 patients / 20 wounds.	Surface swabs from healthy skin and the wound immediately before and after sharp debridement; high-throughput 16S rRNA gene sequencing; differential-abundance and Bayesian association analyses.	A single debridement did not produce a detectable immediate shift in overall wound microbiome composition. Facultative anaerobes, particularly Enterobacter, were associated with failure to heal within 6 months.
**[3] Wolcott et al. (2016)**	Large retrospective, cross-sectional molecular survey.	Mixed chronic wounds, including diabetic foot, venous leg, and pressure/decubitus ulcers.	2,963 patients.	Clinical wound specimens profiled by bacterial 16S rDNA amplicon pyrosequencing; community composition compared across wound categories and available demographic variables.	Chronic wounds were highly polymicrobial. Common genera included Staphylococcus, Pseudomonas, Corynebacterium, and anaerobes; wound type and available patient demographics explained little of the observed compositional variation.
**[8] Radzieta et al. (2021)**	Cross-sectional multiomics pilot study stratified by clinical infection severity.	Clinically infected diabetes-related foot ulcers classified as mild, moderate, or severe using PEDIS criteria.	39 participants enrolled; 36 tissue specimens yielded shotgun-metagenomic data (9 mild, 15 moderate, and 12 severe infections), and 14 specimens yielded RNA-sequencing data.	Tissue punch biopsies divided for conventional culture, shotgun metagenomics, and dual RNA sequencing; taxonomic and functional profiling of microbial reads, microbial metatranscriptomics, host-transcriptome analysis, and integration with clinical and laboratory metadata.	Overall microbial composition differed little across severity groups, but severe infections were more diverse and showed active multispecies virulence programs. Mild-to-moderate infections were often dominated by active S. aureus and Streptococcus, whereas severe infections showed broader host inflammatory, acute-phase, immune, and stress responses with depletion of wound-healing and myogenesis programs.

**[9] Smith et al. (2016)**	Cross-sectional exploratory comparison of newly developed and recurrent ulcers.	Diabetic foot ulcers in adults with type 2 diabetes; new ulcers versus recurrent ulcers.	20 participants / 20 ulcers (10 new and 10 recurrent); 16 ulcer samples yielded interpretable 16S sequencing data (9 new and 7 recurrent).	Ulcer swabs assessed by conventional aerobic/anaerobic culture and 16S rRNA gene amplicon sequencing on an Illumina HiSeq 2500; paired healthy-skin swabs were collected as controls; community diversity and clinical associations, including HbA1c and diabetes duration, were explored.	Sequencing detected a broader, predominantly facultative or obligately anaerobic community than culture. Community composition and diversity did not differ clearly between new and recurrent ulcers; lower HbA1c and shorter diabetes duration correlated with greater within-ulcer diversity.
**[11] Dunyach-Remy et al. (2021)**	Prospective longitudinal pilot cohort.	Pressure ulcers in patients with spinal cord injury undergoing neurological rehabilitation.	24 patients: 15 improved and 9 stagnated/worsened by day 28.	Pressure-ulcer tissue biopsies at baseline and day 28; 16S rRNA V3-V4 amplicon sequencing; alpha- and beta-diversity analyses related to wound evolution.	Overall alpha diversity did not change differently by outcome, whereas community composition (beta diversity) differed according to clinical evolution, supporting an association between microbiota dynamics and wound trajectory.
**[12] Loesche et al. (2017)**	Prospective longitudinal observational cohort with repeated measures for up to 26 weeks.	Neuropathic DFUs managed with standardized wound care.	100 participants	Sampling approximately every 2 weeks; 16S rRNA gene sequencing; Dirichlet-multinomial community typing, Markov-chain transition analysis, and mixed-effects models incorporating healing, antibiotics, and complications.	Temporal stability of the DFU microbiota was associated with poor healing, whereas more frequent community transitions were associated with favorable outcomes. Antibiotic exposure and wound complications increased community disruption without producing a uniform restorative pattern.
**[15] Zhang et al. (2023)**	Secondary metagenomic and bioinformatic analysis of public sequence data.	Tissue from diabetic foot infections (DFIs).	36 tissue metagenomes retrieved from the NCBI Sequence Read Archive	Shotgun-metagenomic read analysis against antimicrobial-resistance databases; characterization of core/accessory resistomes and associations with microbial taxa and mobile genetic elements.	Detected 229 antimicrobial-resistance gene subtypes in 20 resistance classes, including a 24-gene core resistome dominated by multidrug, tetracycline, macrolide-lincosamide-streptogramin, and beta-lactam resistance determinants.
**[26] De Wert et al. (2020)**	Hospital-based cross-sectional case-control study.	Unaffected skin of patients with a sacral pressure ulcer versus hospitalized patients without a	15 pressure-ulcer cases and 15 controls.	Skin swabs from unaffected sites; 16S rRNA V4 amplicon sequencing; diversity, differential-abundance, and random-forest classification analyses.	Patients with pressure ulcers showed greater inter-individual microbiome variation and differential abundance of 23 species; Enterococcus and S. aureus were prominent among taxa associated with pressure-ulcer status.

		pressure ulcer.			
**[35] Kalan et al. (2016)**	Prospective longitudinal observational cohort with serial fungal-community profiling and complementary interkingdom biofilm experiments; follow-up to 26 weeks.	Non-healing diabetic foot ulcers (DFUs) managed under a standardized wound-care protocol.	100 participants / 100 DFUs; 384 serial specimens collected approximately every 2 weeks. Fungal taxa were detected by ITS1 sequencing in specimens from 79 participants.	Pan-fungal ITS1 amplicon sequencing with clinical-outcome and host-factor analyses; comparison with culture and previously generated bacterial 16S profiles; directed culture of selected yeast-bacterium pairs and confocal biofilm imaging.	Fungal communities were prevalent, heterogeneous, and temporally dynamic. Fungal diversity increased with antibiotic exposure and clinical complications; baseline community features and pathogenic fungi were associated with delayed healing and necrosis. Selected fungal and bacterial isolates formed mixed biofilms.
**[36] Wang et al. (2024)**	Cross-sectional comparative metagenomic study of chronic versus non-chronic wound infections.	Clinically heterogeneous wound infections; chronic and non-chronic groups defined by wound duration.	66 patients: 28 with chronic wound infections and 38 with non-chronic wound infections.	Metagenomic next-generation sequencing of wound samples; bacteriome and mycobiome characterization; associations with systemic inflammatory markers; bacteria-fungi correlation-network analyses.	Chronic wound infections showed greater P. aeruginosa abundance. C. glabrata correlated positively with P. aeruginosa, while M. restricta was associated with anaerobic taxa, supporting potential interkingdom ecological relationships.
**[37] Verbanic et al. (2022)**	Prospective observational virome study with paired sampling before and after one sharp-debridement episode and 6-month healing ascertainment.	Heterogeneous chronic wounds, including diabetic, venous, arterial, and pressure wounds; contralateral healthy skin as comparator.	20 patients / 20 wounds, with paired pre- and post-debridement wound samples and contralateral-skin samples; 8 wounds healed and 12 remained unhealed at 6 months.	Virus-like-particle enrichment followed by shotgun sequencing; reference-dependent classification with Kraken2/Bracken against NCBI Viral RefSeq and IMG/VR; ecological diversity and differential-abundance analyses integrated with the previously characterized bacterial fraction.	Wounds contained more diverse viromes than contralateral skin and were dominated by bacteriophages targeting common wound bacteria. Wound and skin viromes were compositionally distinct, and different phage profiles were associated with healed and unhealed wounds.
**[38] Bach et al. (2022)**	Translational mechanistic study combining a prospective human cohort with murine wound-infection models and cellular assays.	Chronic wounds infected with P. aeruginosa; complementary murine infected-wound models and keratinocyte/immune-cell experiments.	36 patients with chronic P. aeruginosa wound infections (25 Pf-positive and 11 Pf-negative); animal and cell-experiment sample sizes varied by assay.	Detection of filamentous Pf phage in clinical P. aeruginosa isolates with longitudinal wound-size/healing assessment; Pf-producing versus Pf-deficient bacterial comparisons in murine wounds; histology, barrier assays, cytokine analyses, and keratinocyte-migration experiments focused on CXCL1 signaling.	Pf-positive infections were more likely to increase in wound size. Mechanistic experiments indicated that Pf impaired re-epithelialization and keratinocyte migration through inhibition of CXCL1 signaling, independently of a simple increase in bacterial virulence.

**[42] Ernlund et al. (2021)**	Prospective paired pre/post cleansing study.	Chronic venous stasis ulcers.	11 patients / 11 ulcers.	Wound swabs immediately before and after washing with hypochlorous-acid- or chlorhexidine-soap-based regimens; 16S rRNA gene sequencing plus culture-based analysis.	Cleansing did not consistently alter community diversity but produced reproducible patient-specific changes in composition, often reducing the relative abundance of the dominant genus; culture supported a reduction in viable bacterial burden.
**[46] Hunter et al. (2020)**	Uncontrolled prospective case series of continuous topical oxygen added to standard care; 8-week follow-up.	Chronic DFUs present for at least 4 weeks.	6 patients / 6 ulcers; 5 healed and 1 did not heal.	Weekly wound swabs during continuous topical oxygen therapy; metagenomic 16S rDNA next-generation sequencing; descriptive longitudinal comparison with wound closure.	The five healed ulcers shifted toward more diverse communities enriched in aerobes/facultative anaerobes, whereas the non-healing ulcer retained an anaerobe-dominated microbiome.
**[47] Malone et al. (2021)**	Single-arm proof-of-concept study of concentrated surfactant gel plus standard care for 6 weeks.	Non-healing DFUs with microscopy-confirmed chronic biofilm infection.	10 patients / 10 ulcers.	Gel applied every 2 days; wound-base swabs at weeks 0, 1, 3, and 6; 16S DNA sequencing and qPCR for composition/load; baseline tissue assessed by scanning electron microscopy and PNA-FISH/confocal microscopy.	Seven of ten ulcers showed a reduction in total microbial load (mean reduction about 0.8 log10 16S copies), while three increased. Dominant taxa changed in an individualized manner; load, composition, and diversity did not change concordantly.
**[51] Gardiner et al. (2017)**	Prospective longitudinal exploratory cohort; six visits over 10 weeks.	Diabetic foot skin and chronic foot ulcers; non-diabetic foot skin as comparator.	8 participants with type 2 diabetes and 8 non-diabetic controls.	Sole skin swabs at 2-week intervals; diabetic wound swabs and debrided tissue when available; bacterial 16S rRNA amplicon sequencing and longitudinal community analyses.	Diabetic foot skin had reduced bacterial diversity relative to controls. Ulcer communities were highly heterogeneous across patients and time, with recurrent shifts in dominant taxa rather than convergence on one universal DFU microbiome.
**[56] Min et al. (2020)**	Prospective exploratory cohort assessing baseline and 1-week response to sharp debridement/offloading, with 4-week healing assessment.	Vascularized neuropathic DFUs; wound bed, wound edge, and adjacent skin compartments.	10 patients; 50 site/time-point samples.	16S rRNA next-generation sequencing of spatially matched samples at baseline and after 1 week of standard care; diversity and taxa-outcome association analyses.	Wound beds contained more distinct phylotypes than edges or adjacent skin. One week of care did not significantly change global diversity/composition. Higher baseline Peptoniphilus abundance was associated with impaired healing in an especially small subset (n=5).

**[58] Malone et al. (2017)**	Prospective observational cohort of new clinically infected DFUs, with treatment outcome follow-up.	Diabetic foot infections sampled by ulcer tissue punch biopsy.	39 patients; 19 experienced antimicrobial treatment failure.	Baseline tissue punch biopsy; 16S rRNA next-generation DNA sequencing and conventional culture; microbiota correlated with ulcer duration, infection severity, laboratory/clinical variables, and treatment outcome.	Ulcers of less than 6 weeks were usually dominated by one pyogenic species, whereas longer-duration ulcers were highly polymicrobial and severe infections had more complex communities. Overall baseline microbiome composition did not distinguish treatment failure from success.
**[59] Mudrik-Zohar et al. (2022)**	Cross-sectional diagnostic-comparison cohort with clinical-outcome association analyses.	Hospitalized patients with infected DFUs; wound tissue biopsy.	31 patients.	Conventional culture compared with 16S rRNA and shotgun-metagenomic sequencing of wound biopsies; taxa profiles related to clinical characteristics and conservative versus surgical outcomes.	Molecular methods detected a broader polymicrobial community and substantially more anaerobes than culture. Gram-negative organisms predominated across methods, while some culture/outcome associations were not reproduced consistently by sequencing.

Supplementary Table S1. Characteristics and key findings of primary studies included in the article. Eligible primary studies were original chronic-wound investigations assessing bacterial, fungal, or viral community composition, diversity, temporal dynamics, resistome features, or clinically relevant phage-host relationships using 16S rRNA gene sequencing, fungal ITS sequencing, shotgun metagenomics, or virus-enriched metagenomic approaches.
